# Predictors of 30-day mortality in cancer patients with septic shock

**DOI:** 10.1186/cc14617

**Published:** 2015-03-16

**Authors:** E Osawa, C Park, F Bergamin, B Pileggi, J Almeida, R Nakamura, I Duayer, G Queiroz, F Galas, J Ribeiro, I Bispo, J Fukushima, L Hajjar

**Affiliations:** 1 Institute of Cancer of University of São Paulo, Brazil

## Introduction

In cancer patients, sepsis is the main cause of admission to the ICU and is associated with elevated mortality rates and healthcare costs. In this population, specific factors such as poor functional status and immunosuppression secondary to malignancy and/or antineoplastic treatment contribute to decreased survival. The aim of this study is to identify predictors of mortality in cancer patients admitted to the ICU with septic shock.

## Methods

This is a retrospective study that analyzed predictors of 30day mortality in 269 patients admitted to the ICU of the Institute of Cancer of State of São Paulo, Brazil, from February 2012 to November 2014.

## Results

From 1,250 patients admitted to the ICU, 269 patients had the admission diagnosis of septic shock and were analyzed. The mean age was 62 ± 14 years and 152 patients (56.5%) were male. Most patients had solid cancer (93.6%), and 87 patients (34.5%) had gastrointestinal neoplasm. Upon admission, the median SOFA score was 4 (IQR: 2 to 7), median SAPS 3 score was 55 (IQR: 48 to 68) and median lactate level was 1.88 mmol/l (IQR: 1.22 to 3.21). After 48 hours of ICU admission, acute kidney injury (AKI) was diagnosed according to AKIN classification as follows: 202 patients (75.1%) had grade 0, 49 (18.2%) grade 1, seven (2.6%) grade 2 and 11 (4.1%) had grade 3. The 30-day mortality rate was 24.9%. In the univariate analysis, associated variables with 30-day mortality were age, urea and creatinine levels at admission, SOFA score at admission, SAPS 3 score and 48-hour AKI. In multivariate analysis, the predictive factors for 30-day mortality were SOFA score at admission (OR = 1.12; 95% CI: 1.04 to 1.21, *P *= 0.002) and 48-hour AKI defined as AKIN grades 1, 2 and 3 (OR = 2.69; 95% CI: 1.45 to 4.97, *P *= 0.002).

## Conclusion

In cancer patients with septic shock, SOFA score at admission and acute kidney injury at 48 hours of admission were predictors of 30-day mortality. These findings reinforce the needing of early strategies of diagnosis and therapy in this subset of patients.

